# P-1064. Beta-Lactams Strike Back: Challenging Resistance Assumptions in Serratia marcescens

**DOI:** 10.1093/ofid/ofaf695.1259

**Published:** 2026-01-11

**Authors:** Caroline L Terrell, Anthony M Casapao, Makayla A Bresnock, Lisa Vuong, Danielle K Sanchez, Yvette McCarter, Carmen Isache, Christopher A Jankowski

**Affiliations:** UF Health, Jacksonville, Florida; University of Florida College of Pharmacy, Jacksonville, FL; UF College of Pharmacy, Jacksonville, Florida; UF Health, Jacksonville, Florida; UF Health, Jacksonville, Florida; UF Health Jacksonville, Jacksonville, Florida; UF Health Jacksonville, Jacksonville, Florida; UF Health, Jacksonville, Florida

## Abstract

**Background:**

The management of serious *Serratia marcescens* (SEM) infections is controversial due to the uncertainty of clinically significant ampC beta-lactamase overexpression. The purpose of this study was to evaluate differences in clinical outcomes in patients with SEM bloodstream infection (BSI) or pneumonia (PNA) who receive ceftriaxone (CRO), piperacillin-tazobactam (TZP), or cefepime (FEP).

**Table 1.**
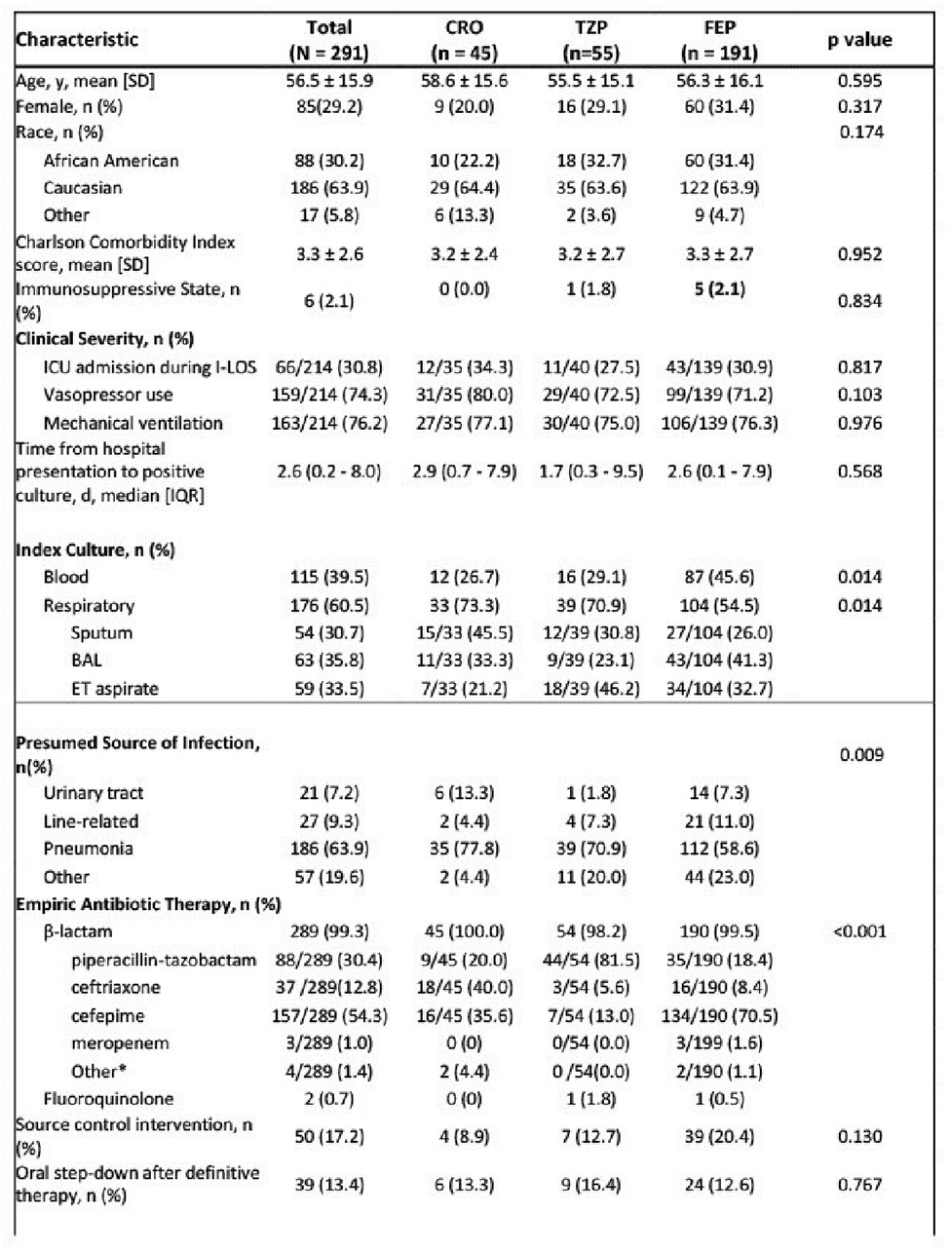
Patient demographics and clinical characteristics of SEM management

**Table 2.**
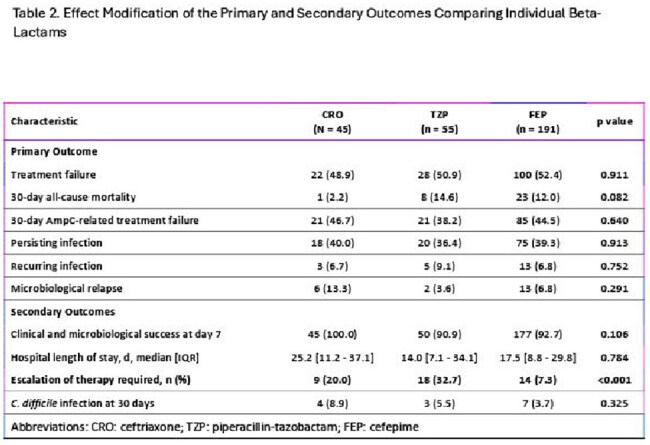
Effect Modification of the Primary and Secondary Outcomes Comparing Individual Beta-Lactams

**Methods:**

This IRB-approved retrospective, multi-center cohort study included adult patients hospitalized with SEM BSI or PNA over a 10-year period at UF Health. Based on the definitive antibiotic regimen, patients were assigned to CRO, TZP or FEP groups. The primary composite endpoint was treatment failure: persistent infection (fever or leukocytosis on day five of antibiotics), recurrent infection, or microbiological relapse. Secondary outcomes included hospital length of stay (LOS) and escalation of therapy.

**Results:**

A total of 291 patients were included in the study and stratified by definitive antibiotic regimen: CRO (n=45), TZP (n=55), and FEP (n=191). Treatment failure did not differ significantly between CRO (48.9%), TZP (50.9%), and FEP (52.4%), p=0.911. There was no difference in median LOS, with 25.2 days (IQR: 11.2 - 37.1) in the CRO group, 14 days (IQR: 7.1-34.1) in the TZP group, and 17.5 days (IQR: 8.8 - 29.8) in the FEP group, p=0.784. A significantly higher proportion of patients in the CRO (20%) and TZP (32.7%) groups required escalation of therapy compared to FEP (7.3%), p< 0.001.

**Conclusion:**

Treatment with CRO or TZP was not associated with increased treatment failure rates in adult patients being treated for SEM BSI or PNA, but were associated with increased incidence of antibiotic escalation.

**Disclosures:**

All Authors: No reported disclosures

